# Chromosome-level genome assembly of *Babesia caballi* reveals diversity of multigene families among *Babesia* species

**DOI:** 10.1186/s12864-023-09540-w

**Published:** 2023-08-24

**Authors:** Akihiro Ochi, Taishi Kidaka, Hassan Hakimi, Masahito Asada, Junya Yamagishi

**Affiliations:** 1 Equine Research Institute, Japan Racing Association, Shimotsuke, Tochigi Japan; 2 International Institute for Zoonosis Control, Hokkaido University, Sapporo, Hokkaido Japan; 3 National Research Center for Protozoan Diseases, Obihiro University of Agriculture and Veterinary Medicine, Obihiro, Hokkaido Japan; 4 Department of Veterinary Pathobiology, School of Veterinary Medicine and Biomedical Sciences, Texas A&M University, College Station, Texas USA; 5 Global Station for Zoonosis Control, GI-CoRE, Hokkaido University, Sapporo, Hokkaido Japan

**Keywords:** Equine babesiosis, *Babesia caballi*, Comparative genomics, Multigene expansion

## Abstract

**Background:**

*Babesia caballi* is an intraerythrocytic parasite from the phylum Apicomplexa, capable of infecting equids and causing equine piroplasmosis. However, since there is limited genome information available on *B. caballi*, molecular mechanisms involved in host specificity and pathogenicity of this species have not been fully elucidated yet.

**Results:**

Genomic DNA from a *B. caballi* subclone was purified and sequenced using both Illumina and Nanopore technologies. The resulting assembled sequence consisted of nine contigs with a size of 12.9 Mbp, rendering a total of 5,910 protein-coding genes. The phylogenetic tree of Apicomplexan species was reconstructed using 263 orthologous genes. We identified 481 *ves1*-like genes and named “*ves1c*”. In contrast, expansion of the major facilitator superfamily (*mfs*) observed in closely related *B. bigemina* and *B. ovata* species was not found in *B. caballi*. A set of repetitive units containing an open reading frame with a size of 297 bp was also identified.

**Conclusions:**

We present a chromosome-level genome assembly of *B. caballi*. Our genomic data may contribute to estimating gene expansion events involving multigene families and exploring the evolution of species from this genus.

**Supplementary Information:**

The online version contains supplementary material available at 10.1186/s12864-023-09540-w.

## Background

*Babesia caballi* is a protozoan parasite (phylum Apicomplexa) known to infect horses, causing equine piroplasmosis. Horses infected with this parasite experience anemia and systemic illness (i.e., fever, lethargy, anorexia, and peripheral edema) [1]. Equine piroplasmosis has a worldwide distribution, being endemic to tropical, subtropical, and some temperate regions. The main vectors of *B. caballi* include tick species of *Dermacentor*, *Haemaphysalis*, *Hyalomma*, and *Rhipicephalus* genera [2]. It is estimated that approximately 90% of the world’s horses are bred in endemic areas [3]. Furthermore, the growing international movement of horses has also raised concerns regarding outbreaks in nonendemic areas.

The *Babesia bovis* genome sequenced in 2007 was the first whole-genome sequence of a species from the *Babesia* genus [4], followed by the release of the *B. microti* genome [5]. Both genomes were sequenced using the whole-genome shotgun Sanger sequencing approach, being well assembled and containing few gaps. Whole-genome sequences of *B. bigemina, B. divergens, B. ovata,* and *Babesia* sp. Xinjiang species are nowadays also available [6–8]. However, despite next-generation sequencing technologies, assembled genomes in these species showed more fragmentation than *B. bovis* and *B. microti* genomes (Table 1).

**Table 1:** comparative analysis of genome and gene among representative apicomplexan parasites

	*B. caballi*	*B. bigemina*	*B. ovata*	*B. bovis*	*B. sp. Xinjiang*	*B. divergens*	*B. microti*
genome size (bp)	12,816,698	13,840,936	14,453,397	8,179,706	8,373,550	8,915,963	6,392,438
N50 (bp)	3,243,686	2,541,256	2,090,503	1,797,577	533,301	1,092,625	1,766,409
# of contigs/scaffolds	9	483	91	14	215	82	6
# of coding genes	5,910	5,079	5,031	3,706	3,066	4,129	3,494
# of tRNA	65	46	64	69	40	NA	44
# of 5.8S rRNA	9	6	6	9	NA	NA	2
# of 18S rRNA	4	3	3	3	NA	NA	2
# of 28S rRNA	3	3	4	3	NA	NA	2
reference	this study	ref. 6	ref. 7	ref. 4	ref. 8	ref. 6	ref. 5

The phylogenetic relationships between *B. caballi* and related *Babesia* species are still inconsistent. In a previous study, based on 18S rRNA and *cob* sequences, *B. bovis* and *B. bigemina* were located outside of *B. caballi* [9]. In contrast, in another study based on *β-tubulin* and *cox3* sequences, *B. caballi* and *B. bigemina* located in the same clade and *B. bovis* appeared outside of them [9]. Another phylogenetic analysis using *cox1* and *cytb* sequences was also conducted [10] but more genes are required to reach a consensus.

Another inconsistency is observed from a morphological point of view. It is known that there are the ridge structures on the surface of *B. bovis-*infected erythrocytes and they are involved in cytoadhesion and capillary sequestration [10–12]. In contrast, neither ridge structures nor sequestration are observed for *B. bigemina* [13]. In *B. caballi,* no ridge structures in parasitized erythrocytes are observed [14], but sequestration is believed to be involved in persistent subclinical infection [1]. Interestingly, tubular structures in infected erythrocytes of *B. caballi* can be identified by electron microscopy [15]; however, specific molecular characteristics of these structures are unknown. Variant erythrocyte surface antigen 1 (VESA1), initially identified in *B. bovis* [16], is a heterodimeric protein encoded by *ves1α* and *ves1β* genes, which comprise the largest multigene family in *B. bovis* [17, 18]. Proteins encoded by *ves1α* and *ves1β* generally have a cysteine- and lysine-rich domain (CKRD) motif and a c-terminal transmembrane domain [17, 18]. It has been shown that *ves1α* and *ves1β* are mutually transcribed at a genomic location referred to as locus of active transcription (LAT) [19]. The VESA1 of *B. bovis* is known to be involved in cytoadhesion and pathogenicity [12, 20]. Sequence homology and Hidden-Markov-Modeling (HMM) analyses have been applied to identify *ves1a, ves1b,* and *ves2* genes in *B. bigemina* and *B. ovata* from the *B. bovis ves1* gene [6, 7]. Additionally, *ves1* and *ves2* in *B. divergens* and other VESA coding genes in *Babesia* sp. Xinjiang have been identified [6, 8]. Interestingly, *ves*-like genes in the *B. microti* genome have not been detected [5]. In *Babesia* species, VESA likely functions in immune evasion through antigenic variation and plays a role in pathogenicity, although this hypothesis needs further confirmation.

The *multi-transmembrane* (*mtm*) family is another important expanded gene family which has recently been identified to encode proteins with eight or more transmembrane domains [21, 22]. In *B. bovis*, there are two *mtm* sub-families, A-type and B-type. Although the function of *mtm* genes is not yet well understood, it has been found that *mtm* expression is associated with the resistance to the antibiotic blasticidin S. Therefore, it has been suggested that *mtm* genes may be involved in the transport of specific substrates [22]. The *major facilitator superfamily* (*mfs*) is also an expanded gene family encoding multi-transmembrane proteins. In *B. bigemina* and *B. ovata* approximately 40 *mfs* genes have been identified [7, 21, 22]*.* Moreover, in *B. bovis* and *B. divergens,* two *mfs* genes have been detected, while no *mfs* genes have been identified in *B. bigemina* and *B. ovata* genomes*.* Similarly to the *mtm* family, the function of *mfs* genes remains to be elucidated.

Long-term cultivation of *B. bovis* causes quasi species in the population which hamper genome assembly (data not shown). Therefore, we developed a subclone, USDA-D6B2, and applied a hybrid sequencing approach using nanopore long reads and Illumina accurate reads to assemble at the chromosome level. It will contribute to further understanding of evolution, adaptation and parasitic capability of the parasite.

## Results

### *De novo* assembly of *B. caballi* genome

Using Oxford Nanopore and Illumina sequencing, we generated 3.89M (5.14 Gbp) long reads and 8.90M (2.67 Gbp) short paired-end reads, respectively. These reads were assembled into nine contigs covering a total of 12,916,698 bp, which also include apicoplast and a mitochondrial genomes (Table 1). The BUSCO completeness analysis revealed that the genome assembly of *B. caballi* contained 96.8% (432/446) complete, 1.1% (5/446) fragmented, and 2.1% (9/446) of the missing orthologous genes expected to be present in other *Babesia* species, supporting the idea that missing regions were limited. We detected a telomeric sequence pattern with (TTTAGGG)n. From all nine contigs, three contigs, BcabD6B2_scf02, 04 and 05, showed the telomeric sequence pattern at both terminal ends, and three contigs displayed this sequence at one end, thereby suggesting that BcabD6B2_scf02, 04 and 05 contained completely sequences chromosomes. Moreover, contigs corresponding to apicoplast and mitochondrial genomes did not show telomeric sequences.

### Gene model prediction

In total, 5,910 protein coding genes were predicted with AUGUSTUS and then validated using transcriptomic data (Table 1). The average and median coding sequence (CDS) lengths were 1622.7 bp and 1173 bp, respectively. Approximately 74.8% of the *B. caballi* genome consists of CDSs. *B. caballi* contains 47 and 18 tRNA genes scattered on nuclear (chromosomes) and apicoplast genomes, respectively (Table 1). Moreover, nine 5.8S, four 18S, and three 28S rRNA genes were identified (Table 1). We also detected three intact 18S-5.8S-28S rRNA ribosomal *loci*.

### Phylogenetic analyses

**Fig. 1:**
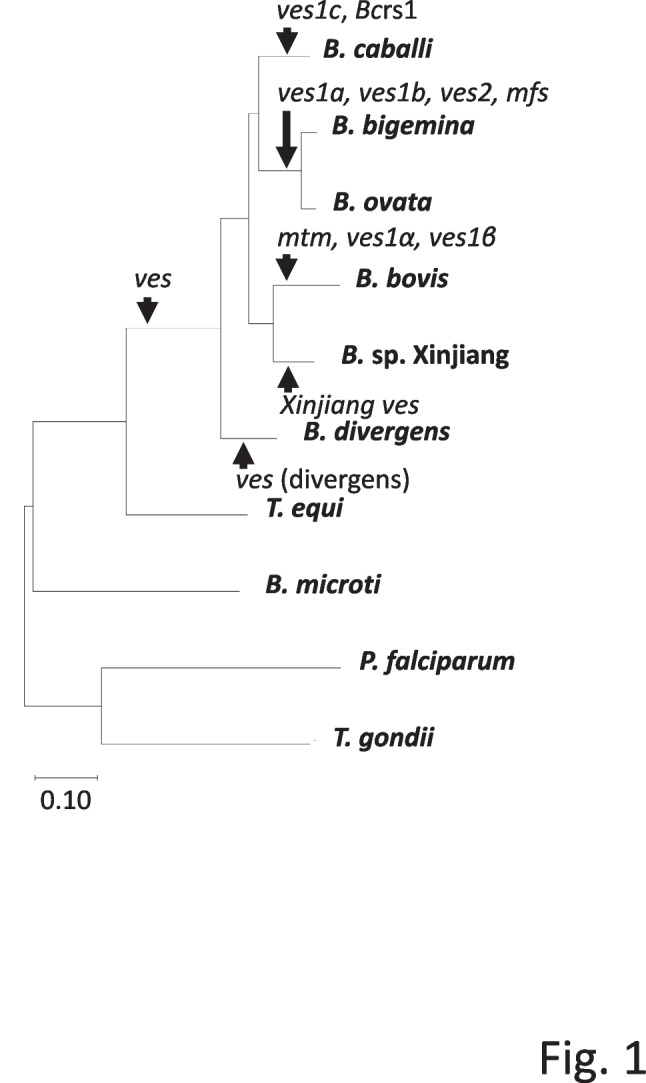
Orthologous-gene-based phylogenetic analysis. The phylogenetic tree was constructed using 263 orthologous genes conserved among *B. caballi, B. bovis, B. bigemina, B. ovata, B. microti, B. divergens, Babesia* sp. Xinjiang*, P. falciparum,* and *T. gondii.* The arrowheads represent estimated expansion events for each gene family and the de novo emergence of repetitive sequences

We generated a phylogenetic tree using 263 orthologous genes which were identified in ten different species (i.e., *B. bovis, B. bigemina, B. ovata, B. microti, B. divergens, Babesia* sp. Xinjiang*, T. equi, P. falciparum, T. gondii,* and *B. caballi*)*.* To construct the phylogenetic tree, orthologous gene sequences were translated, and aligned amino acid sequences were concatenated (Fig. 1). The genome-wide phylogenetic tree indicated that *B. caballi* was closer to *B. bigemina* and *B. ovata* than *B. bovis* and *Babesia* sp. Xinjiang.

Subsequently, a phylogenetic analysis based on apicoplast genomes from *B. caballi*, *B. bovis*, *B. orientaris*, *B.* sp. Xinjiang, *B. ovata*, *B. gibsoni,* and *B. microti* was also performed. First, dotplots were used to verify that no recombination occurred among apicoplast genomes (Fig. S1). The topology of the apicoplast-based phylogenetic tree was inconsistent with the results of the phylogenetic analysis using orthologous genes (Fig. S2).

The V4 regions of the three 18S rRNA genes in the D6B2 *B. caballi* strain were clustered with representative sequences from Clade A1 (Fig. S3).

We also examined the presence and absence of orthologous genes in the *B caballi* genome. From this analysis, 33 and 316 lost genes were found (Table S1). The functional enrichment analysis of orthologous genes was performed, but no significant enrichment was found.

### Multigene families

**Fig. 2:**
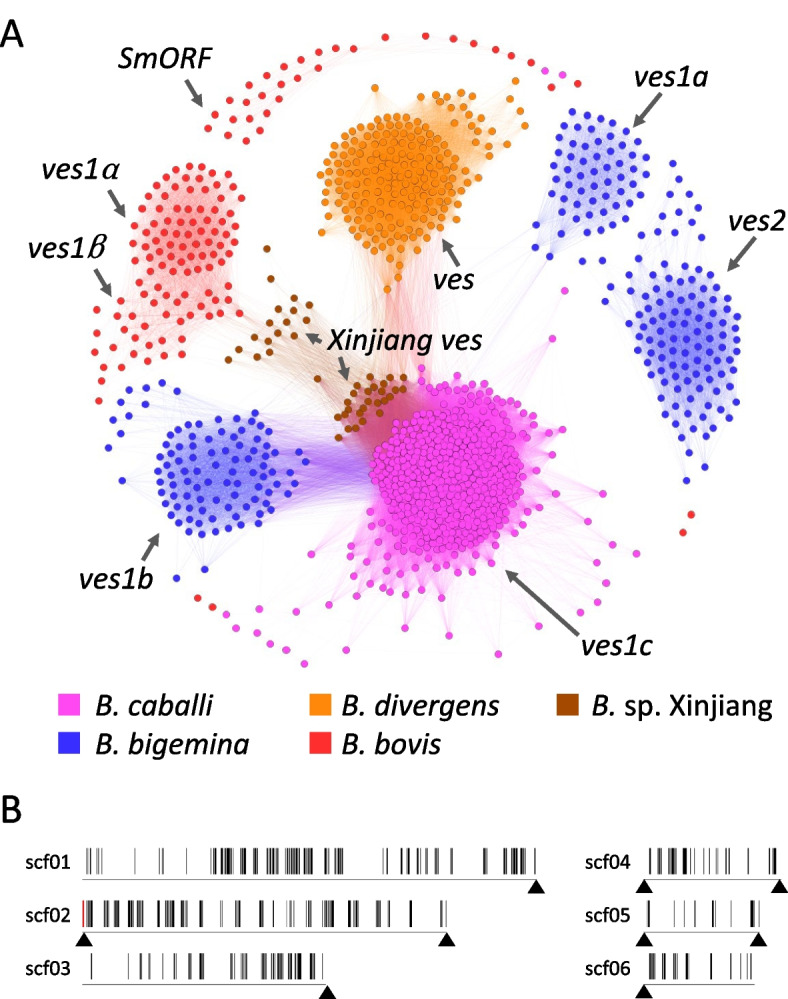
Characterization of *ves1c* genes in *B. caballi*. **A** Sequence homology cluster analysis based on sequence similarity among *ves*-related genes of *B. caballi, B. bigemina, B. divergens, B. bovis, and Babesia* sp. Xinjiang*.* Each node represents a protein-coding gene in the five parasites species analyzed. The edges represent similarity between nodes. **B** Distribution of *ves1c* genes in *B. caballi* genome. The vertical lines represent *ves1c loci*. The horizontal lines represent contigs and their corresponding ID. The *locus* of the exclusively expressed *ves1c* gene, BcabD6B2_18120, is highlighted in red. Telomeric repeats are indicated by arrowheads

The HMM model identified 481 *ves1*-like genes in the *B. caballi* genome (Table S2). Homology between *ves1*-like genes of *B. caballi*, *ves* and *small open reading frame* (*smorf*) genes of *B. bovis, B. bigemina, B. divergens,* and *Babesia* sp. Xinjiang was verified, showing that *B. caballi ves1*-like genes were clustered separately from other *Babesia ves* genes (Fig. 2A). Nonetheless, we found certain similarity between *B. caballi ves1*-like and *B. bigemina ves1b* genes; therefore, we named *B. caballi ves1*-like genes as *ves1c.* These *ves1c* genes shared sequence similarity with several *Babesia* sp. Xinjiang *ves* and *B. divergens ves1* genes; however, no significant similarity to *B. bovis ves1α*, *ves1β*, and *smorf* genes or *B. bigemina ves1a* and *ves2* genes was detected in the analysis*.* For further validation, sequence alignment and phylogenetic analysis were performed for *B. caballi ves1*-like genes; however, no alignment remained after removing the gap. Transcriptome of *ves1c* genes showed that the number of mapped read to one of the *ves1c* genes, BcabD6B2_18120, accounted for 38% of all reads mapped to the 481 *ves1c* genes. It was located at the terminal of contig 2 (Fig. 2B). The second and third most abundant genes were BcabD6B2_30960 and BcabD6B2_10260, accounting for 9% and 8% of all reads for *ves1c* genes, respectively.

*Smorf* is another multigene family initially identified in *B. bovis*. We examined whether there are any homologous genes in the *B. caballi* genome by using blastp against 44 *smorf* genes in *B. bovis*. The most similar gene was BcabD6B2_03440, showing only 22.2% sequence identity and an e-value of 2.37E-05. The HMM model did not identify any *B. caballi* sequence related to *smorf* genes of *B. bovis*. (Fig. 2A).

**Fig. 3:**
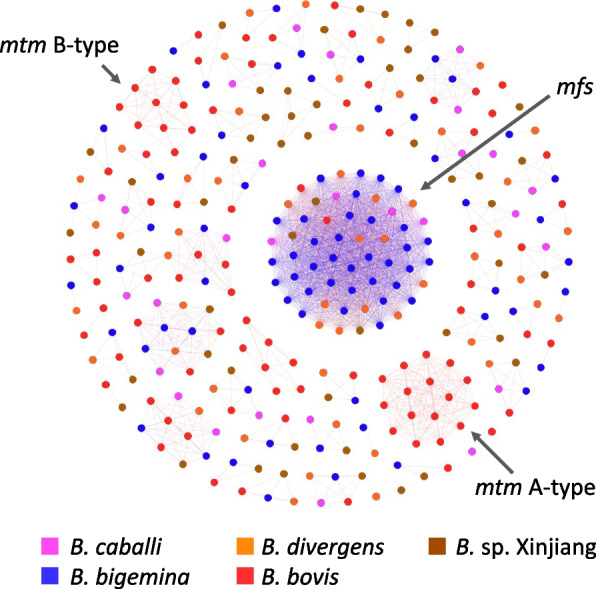
Sequence homology cluster analysis of genes with more than eight transmembrane domains in *B. caballi, B. bigemina, B. divergens, B. bovis, and Babesia* sp. Xinjiang*.* Nodes and edges represent a genes and sequence similarity between nodes, respectively

A total of 29 predicted genes encoding proteins with eight or more predicted transmembrane domains were identified in the *B. caballi* genome. The number of multi-transmembrane protein-coding genes identified in *B. bovis, B. bigemina, B. divergens,* and *Babesia* sp. Xinjiang genomes were 98, 85, 55, and 56, respectively. Therefore, our results suggest that the *B. caballi* genome has fewer genes encoding multi-transmembrane proteins than other *Babesia* species. While predicted multi-transmembrane genes of *B. bovis*, *B. bigemina, B. divergens,* and *Babesia* sp. Xinjiang grouped in the same cluster, no specific clustering pattern was observed for *B. caballi* multi-transmembrane protein-coding genes (Fig. 3).

**Fig. 4:**
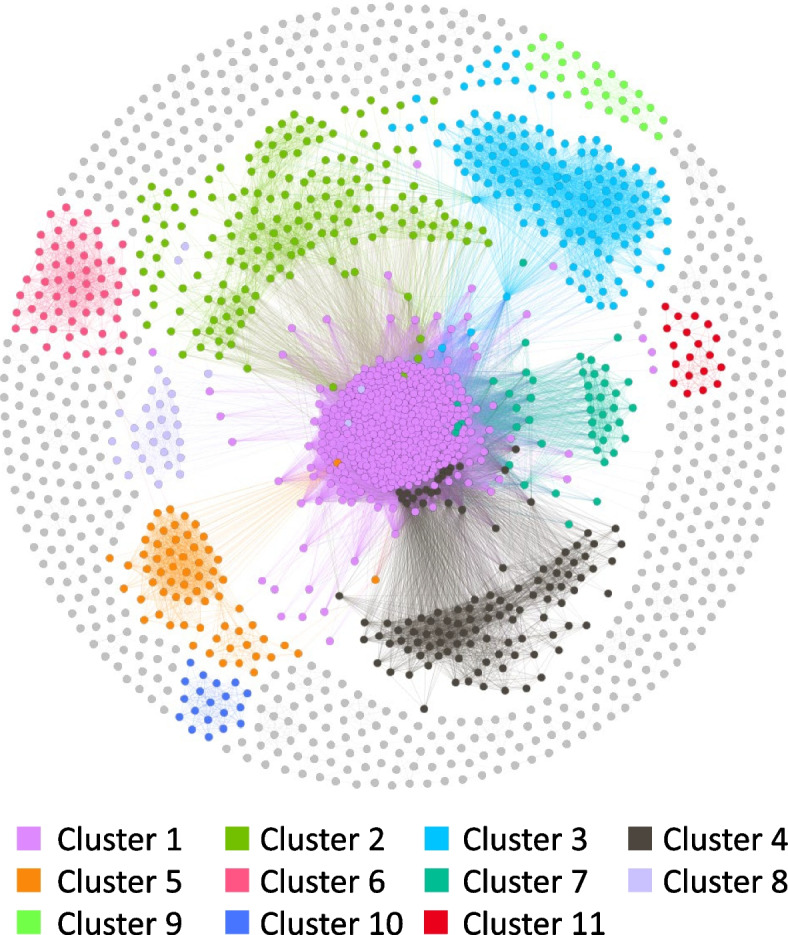
Sequence homology cluster analysis of *B. caballi* genes*.* Nodes and edges represent genes and sequence similarity between nodes, respectively

Other multigene families were searched for similarity against the predicted *B. caballi* genes (Fig. 4 and Table S2). The biggest cluster was cluster 01, which consisted of most *ves1c* genes identified. Moreover, Delta-BLAST revealed that 393 out of the 450 genes in cluster 01 have a VESA1 N-terminal (gnl|CDD|315457) (Table S3A). Among the 450 genes, we identified 259 genes similar to a *Babesia* sp. Xinjiang *ves* gene, BXIN_1821 (Table S3B). In addition, other *Babesia* sp. Xinjiang *ves* genes also shared high similarity levels with *ves1c* genes and even *ves* genes of *B. bigemina, B. bovis, and B. divergens* shared certain sequence similarity (Fig. 2A). Cluster No. 02, 04, 05, 07, and 08 also contained genes with VESA1 N-terminal (gnl|CDD|315457); however, no corresponding clusters in other *Babesia* species were found (Table S3A), but most genes were not identified as *ves*-like genes. Cluster 03 contained genes with a putative reverse transcriptase motif (Table S3A). A similar expansion event involving genes encoding a putative reverse transcriptase motif in *B. ovata* genome has also been reported [7]. Expansion of clusters No. 06, 09, 10, and 11 were unique in *B. caballi;* although their functions were unclear. For further validation, sequence alignment and phylogenetic analysis were performed for genes in each cluster; however, no alignment remained after removing the gap for clusters 01 to 06 and 11.

### Repeat sequences

**Fig. 5:**
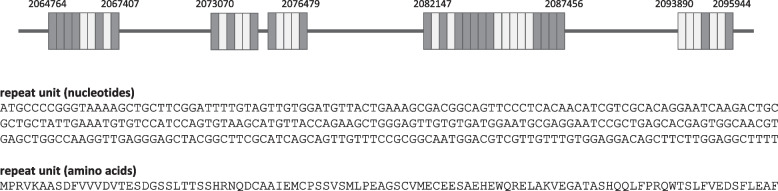
Schematic representation of BcRS1. The light gray boxes represent intact ORFs. The dark gray boxes represent disrupted ORFs with frameshift or truncated site. Numbers represent nucleotide positions in the contig, BcabD6B2_scf02. Nucleotide and encoded amino acids of the repetitive unit are shown at the bottom

Tandem repeat sequences of the *B. caballi* genome were examined using Tandem Repeats Finder. One of the most significant ones was found in the contig, BcabD6B2_scf02, at a position from 2,073,070 bp to 2,095,944 bp (Fig. 5). The repetitive unit consisted of 297 bp with an open reading frame (ORF) encoding 99 amino acids. The largest repeated sequence consisted 17.9 repetitive units, with some having frameshift mutation, while the largest ORF encoded 599 amino acids. Genomic regions with 8.9, 6.9, 5.9, and 4.4 repetitive units were also identified. The analysis of *B. bovis, B. bigemina, B. ovata, B. divergens, B. microti* and *Babesia* sp. Xinjiang genomes did not identify homology to this repeat sequence. The repeat unit was found to fall into cluster 11 (Fig. 4 and Table S3). Other repeat sequences scattered throughout the genome were also examined using RepeatScout (Table S4). Most of the identified repeats were *ves*-related. The tandem repeats found above partially overlapped with *R* = 122. Sequences potentially coding reverse-transcriptase were found, consistent with cluster 3 observed in the sequence homology cluster analysis (Fig. 4 and Table S3). The most abundant repeat sequence was R = 8, covering 707 Kbp across the genome. It consisted of 4.5 repetitions of approximately 1,750 bp units encoding up to approximately 200 amino acids of unknown function.

## Discussion

Using a hybrid sequencing approach, we obtained nine contigs capable of reconstructing almost all complete chromosomes of *B. caballi* (Table 1). Indeed, three contigs exhibited telomeric sequences at both terminal ends, suggesting completed end-to-end sequencing of whole chromosomes (Fig. 2). The BUSCO analysis also supported high integrity in the sequenced and assembled contigs. The long-read sequencing technique likely contributed to this good performance. In addition, sub-cloning might also have helped a successful assembly process since the draft genome of *B. ovata* prepared without this step seemed to be poorly assembled even though long-read sequencing was also conducted [7]. In this regard, we observed that the genetic diversity of *B. bovis* progressively increased during in vitro culture, likely due to segmental gene conversion (unpublished data).

Previously, a phylogenetic study based on*β-tublin* and *cox3* genes inferred that *B. caballi* and *B. bigemina* form a clade separated from *B. bovis* [9]. In contrast, another study using *18S rRNA* and *cob* genes and combined *cox1* and *cytb* sequences indicated *B. bigemina* and *B. bovis* form a clade separated from *B. caballi* [9, 10]. To provide a comprehensive phylogenetic analysis of the relationship between *B. caballi* and other species from the *Babesia* genus, we constructed a phylogenetic tree using 263 orthologous genes. Our results indicated that *B. caballi* was phylogenetically closer to *B. bigemina* than *B. bovis* (Fig. 1). We further examined this phylogeny using apicoplast genome sequences. Firstly, we performed a dotplot analysis to confirm the absence of specific structural variation in the *B. caballi* apicoplast genome (Fig. S1). Subsequently, we observed that the topology of the phylogenetic tree based on apicoplast genomes differed from that using concatenated orthologous gene sequences. However, both phylogenetic trees supported the notion that *B. caballi* is closer to *B. ovata* than *B. bovis* or *Babesia* sp. Xinjiang (Fig. 1 and Fig. S2). Collectively, our results are not conclusive on the evolutional history of *Babesia* species, but the phylogenetic tree using orthologous genes seems to be more consistent because it was constructed using 263 gene sequences encoding 118,671 amino acids scattered throughout the genome and phylogenetic tree based on the apicoplast genomes was constructed by much shorter nucleotides alignment [4, 6, 7]. Genetic diversity among *B. caballi* strains/isolates has been examined using 18S rRNA gene sequences [23–25]. Those studies differ in their nomenclature of clusters, namely alphabetical (A, B, C) or numerical (1, 2, 3). Moreover, it has been shown that the V4 hypervariable region can discriminate clades at a high resolution [26]. We followed this approach and found the *B. caballi* genome encoded three set of 18S rRNA genes clustered together in Clade A1. This is consistent with the observation that Clade A1 is distributed worldwide, including Florida (USA), where the USDA strain used in this study was isolated.

It is known that Apicomplexan parasites have different telomeric sequence patterns [27]. For instance, *Cryptosporidium parvum*, *Theileria annulata*, and *B. bovis* exhibit TTTAGG, TTTTAGGG, and TTTAGGG, respectively. Our our results revealed that telomeric sequences are conserved between *B. caballi* and *B. bovis*, suggesting that telomeric patterns may also be evolutionarily conserved in *Babesia* species; however, more evidence is required to support this hypothesis.

We estimated that the *B. caballi* genome contains 5,910 protein-coding genes. It was approximately more than 800 genes than closely related *B. bigemina* and *B. ovata* [6, 7]. The well-assembled genome sequence obtained in this study likely contributed to this difference. Moreover, the expansion of *ves* genes in *B. caballi* genome might also be another factor associated with the high number of protein-coding genes.

The variant erythrocyte surface antigen 1 (VESA1) heterodimeric protein complex is encoded by a multigene family originally identified in *B. bovis* [16]. It has been shown that other *Babesia* species, including *B. bigemina* and *B. ovata, B. divergens,* and *Babesia* sp. Xinjiang, encode *ves*-related genes [6–8]. In this study, we identified 481 putative *ves*-like genes (Table S2). Sequence homology revealed that *B. caballi ves*-like genes formed a well-differentiated cluster with sequence similarity to *B. bigemina ves1b* genes (Fig. 2A). Therefore, we named this novel gene family “*ves1c”*. We further performed alignment and phylogenetic analysis for the *ves1c* genes; however, no sequence alignment remained after removing gaps. It suggests a high diversity not only among *ves*-genes of different species, but also among the same species. Our transcriptome analysis revealed that the transcripts of BcabD6B2_18120 gene accounted for 38% of total transcripts of *ves1c* genes, suggesting that transcriptional regulation of *B. caballi ves1c* genes is similar to that in *ves1* genes of *B. bovis* and *B. bigemina* [4, 6]. Interestingly, the most abundantly transcribed *ves1c* gene, BcabD6B2_18120, were located in the telomeric vicinity of the contig 2 (Fig. 2B).

We identified several repetitive sequences, being the most representative found in the contig, BcabD6B2_scf02, at a position from 2,073,070 bp to 2,095,944 bp. We named this repetitive sequence “*B. cabali* repetitive sequence 1 (Bcrs1)” (Fig. 5). Bcrs1 has a size of 297 bp encoding 99 amino acids, which are arranged in a tandem array containing almost 44 units, i.e., including both intact and incomplete units. The function of Bcrs1 is unknown since it has homology with neither Apicomplexan nor other species sequences. Therefore, Bcrs1 is also a promising target for species-specific sequence identification.

The occurrence of gene expansions during the evolution of *Babesia* species could be inferred by integrating phylogenetic information (Fig. 1). The *ves* comprise a unique gene family present in all member of the *Babesia* genus except *B. microti.* The *ves1c, ves1b, and Xinjiang ves* shared certain similarity (Fig. 2), suggesting that they evolved from the same ancestral genes. On the other hand, *ves1a* and *ves2* genes likely underwent an expansion after the divergence between *B. caballi* and the ancestral species of *B. bigemina* and *B. ovata.* The *mtm* genes form another gene family characterized to have eight or more transmembrane domains. The *mtm* gene family includes several sub-family, i.e., A-type and B-type (only identified in *B. bovis*) and the *mfs-*expanded-type in *B. bigemina* and *B. ovata*. However, *mfs* genes were absent in *B. caballi* (Figs. 1 and 3), suggesting gene expansion after the speciation process that originated *B. caballi* and before the divergence of *B. bigemina* and *B. ovata.* It is also suggested that the repetitive expansion of Bcrs1 occurred after the speciation because Bcrs1 is present only in *B. caballi* but absent in *B. bigemina* and *B. ovata* (Fig. 1). We hypothesize that this type of species-specific gene expansion event could be associated with host specific parasite adaptation.

## Methods

### Obtention of the *B. caballi* strain and culture

The *B. caballi* USDA strain was isolated from ticks in Florida, the USA in the 1960s and maintained in horses by serial passage of blood [28]. The *B. caballi*-infected horse blood was obtained from U. S. Department of Agriculture (Ames, Iowa). The parasite was isolated as previously described [29], and cultured in vitro in RPMI1640 medium (Sigma-Aldrich, Tokyo, Japan) containing 40% horse serum, 13.6 µg/ml hypoxanthine (Sigma-Aldrich), 1% GlutaMAX-I (Sigma-Aldrich), and 10% horse red blood cells (RBCs). We developed the USDA-D6B2 subclone, which derived from two rounds of limiting dilution.

### Genomic DNA extraction, library construction, and sequencing

*B. caballi*-infected RBCs were treated with saponin to remove hemoglobin and the genomic DNA was then extracted by the Wizard Genomic DNA Purification Kit (Promega), following the manufacturer’s instructions. The library was constructed using the Ligation Sequencing Kit, SQK-LSK109 (Oxford Nanopore Technologies) and sequenced using a FLO-MIN107 flowcell (Oxford Nanopore Technologies). The Fast5 files generated by MinION were basecalled with guppy_basecaller: version 3.2.4 + d9ed22f (Oxford Nanopore Technologies). A PCR free library with 350-bp was constructed using the NEBNext Ultra IIDNA Library Prep Kit (NEB) provided by Novogene Ltd and 150 bp paired-end sequences were obtained using the Illumina NovaSeq 6000 platform (Illumina).

### RNA-seq analysis

Total RNA was extracted from in vitro cultured *B. caballi* parasites using TRIzol reagent (Sigma), following the manufacturer’s instructions. The quality and quantity of the purified RNA were assessed using a Bioanalyzer (Agilent). The library was constructed using the TruSeq Stranded mRNA LT Sample Prep Kit (Illumina), and 300 bp paired-end reads were sequenced using Miseq (Illumina). The obtained reads were aligned with TopHat2 v2.1.1 [30] and counted using HTSeq 2.0.2 [31].

### *De novo *genome assembly

The MinION long reads of *B. caballi* were assembled using Canu version 1.5 based on the following parameters: genomeSize = 10m -nanopore-raw ovsConcurrency = 80 [32]. The resulting contigs were polished using the Illumina reads by pilon version 1.22 [33]. A total of seven iterations were applied until results remained constant. Apicoplast genome was identified using Blast against the *B. bovis* apicoplast genome. It was visualized by YASS [34] then manually circularized. The mitochondrial genome was not found in the original genome assembly. A second assembly was constructed by abyss-pe assembler from the ABYSS program v2.1.5 [35] using the Illumina reads based on k = 128 parameter. The mitochondrial sequence was identified by Blast against the *B. bovis* mitochondrial genome.

### Gene model estimation and functional annotation

Protein-coding gene models were estimated with AUGUSTUS version 3.3.3 [36] using the RNA-seq data mentioned above. Trained parameters were obtained by webAugustus [37] using a *B. bovis* genome sequence (PiroplasmaDB-5.1_BbovisT2Bo_Genome.fasta) and a full-length EST set (B.bov.FL-EST.fa) retrieved from PiroplasmaDB and DB-AT, respectively [38, 39]. The tRNAs and rRNAs were annotated using tRNAscan-SE 2.0 [38] and barrnap version 0.8, respectively. The functional annotation of protein-coding genes was performed with blast2go [39]. Gene models for the apicoplast and the mitochondria genomes were predicted with DFAST [40].

### Acquisition of publicly available sequences and gene annotation

For comparative genomic analysis among Apicomplexan parasites, we used genome assembly in fasta format and annotation in general feature format (gff) available at the PiroplasmaDB database, i.e., obtained from PiroplasmaDB-37 (*B. bovis* T2Bo strain), PiroplasmaDB-36 (*B. bigemina* BOND strain), PiroplasmaDB-40 (*B. ovata* Miyake strain and *B. microti* RI strain), PiroplasmaDB-46 (*B. divergens* 1802A strain), PiroplasmaDB-55 (*Babesia* sp. Xinjiang)*,* PiroplasmaDB-55 (*T. equi*), ToxoDB-40 (*T. gondii* ME49 strain), and PlasmoDB-56 (*P. falciparum* 3D7 strain).

### Phylogenetic analyses

An orthology inference analysis was conducted by the OMA package (v2.5.0) [41] with default parameters. Putative orthologous genes encoded by the genomes of the parasitic species specified before were selected. Amino acid sequences of these orthologous were aligned using MAFFT [42] with default parameters. After the gaps were trimmed, the remaining sequences were concatenated. We then constructed a phylogenetic tree based on the maximum likelihood method with MEGA version 10.0.5 [43]

The structural variation among *B. caballi* (BPLF01000008), *B. bovis* (NC 011395), *B. orientaris* (KT 428643), *Babesia* sp. Xinjiang (KX881914), *B. ovata* (BDSA01000044), *B. gibsoni* (MN481613), and *B. microti* (LK028575) apicoplast genomes were validated. First, genome positions were sorted using the 50S ribosomal protein L2 gene, and dotplots were generated with YASS [34]. Subsequently, phylogenetic relationships among apicoplast genomes were also examined.

A phylogenetic tree was constructed using three 18S rRNA *loci* (BcabD6B2_91080, BcabD6B2_91090, and BcabD6B2_91140) and representative sequences were used for intra-species [26] and inter-species analysis [9]. A total of 68 sequences were aligned with MUSCLE [44] in MEGA version 10.0.5 [43], and a conserved region of 247 bp length comparable to the V4 region was selected to construct a phylogenetic tree by the neighbor-joining method [45].

### Uniquely gained and lost genes in *B. caballi*

The uniquely gained genes in the *B. caballi* genome were defined as those t with no orthologous sequences in *B. bovis*, *B. bigemina*, *B. ovata, B. divergens,* or *Babesia* sp. Xinjiang genomes*.* A gene ontology (GO) enrichment analysis of uniquely gained and whole-predicted *B. caballi* genes was performed using agriGO [46]. The uniquely lost genes were defined as the genes conserved in the five *Babesia* species mentioned above but with no orthologous in *B. caballi. B. bovis* genes retrieved from the PiroplasmaDB database were used as representatives in GO enrichment and metabolic pathway enrichment analysis.

### Identification of multigene families

The members of *ves* and *smorf* multigene families in the *B. caballi* genome were searched using Hidden Markov Model. The training data set was constructed using publicly available genomes and annotations mentioned above. Specifically, *ves1α*, *ves1β*, and *smorf* genes from *B. bovis* were retrieved from gff annotation files by searching “variant erythrocyte surface antigen-1, alpha”, “variant erythrocyte surface antigen-1, beta”, and “*smorf*”, respectively. *Babesia divergens ves1* genes were retrieved from the gff by searching “variant erythrocyte surface antigen”. *Babesia bigemina ves1a, ves1b, ves1ba,* and *ves2* genes were retrieved from the Sanger database (https://www.sanger.ac.uk/resources/downloads/protozoa/babesia-bigemina.html). *Babesia xinjiang ves* were retrieved from a previous study [8]. Hidden Markov Models for each gene set were constructed, and *ves*-related genes in *B. caballi* genome were identified using HMMER version 3.3.2 [47]. *Babesia caballi* genes and *ves* and *smorf* genes in *B. bovis, B. divergens, B. bigemina,* and *Babesia* sp. Xinjiang genome were aligned using BLASTP [48]. We selected genes showing a bit-score higher than 200, and mutual similarity was visualized using the Gephi by a Fruchterman–Reingold layout [49].

To identify proteins having equal or more than eight predicted transmembrane domains and at least one TM domain per 100 amino acids, the multi-transmembrane gene family in the *B. caballi* genome was examined using TMHMM version 2.0c [50]. The same methodology was applied in *B. divergens* and *Babesia* sp. Xinjiang genome. Genes from the multi-transmembrane family in *B. bovis* and *B. bigemina* genomes have been retrieved from a previous study [22]. Sequence similarity among genes was examined and visualized using the method mentioned before.

To find other multiple gene families, genes in the *B. caballi* genome were aligned to themselves using BLASTP. Genes with a bit-score higher than 200 were selected, and mutual sequence similarity was visualized using Gephi by a Fruchterman–Reingold layout [49]. Clusters were assigned by the modularity algorithm of Gephi. The function of the gene sets were estimated using BLASTP with the NR database and Delta-BLAST for functional motifs in the CDD database provided by NCBI.

### Identification of repetitive and repeat sequences

Repetitive sequences in the *B. caballi* genome were searched with Tandem Repeats Finder version 4.09 based on the parameters: 2 7 7 80 10 50 500 -f -d -m [51]. Repeated sequences were also searched using RepeatScout based on the parameter: -l 14 [52]. Blastn was used to verify if predicted sequences included CDSs. The retrieved DNA sequences translated into amino acids in six frames, and functional motifs were searched using Pfam [53].

### Supplementary Information

**Additional file 1: Table S1.** Uniquely gained and lost genes in B. caballi. **Table S2.** Clustered genes in B. caballi. **Table S3A.** Estimated function of set of genes in each cluster by delta-blast for functional motif identification. **Table S3B.** Estimated function of set of genes in each cluster by blastp for homologus gene identification. **Table S4.** Repeat sequences.

**Additional file 2: Fig. S1.** Structural comparison among apicoplast genomes of Babesia species. The alignments among apicoplast genomes of seven Babesia species are shown using dotplots. **Fig. S2.** Phylogenetic analysis using apicoplast genomes of seven Babesia species. The genomes were aligned, and gaps were trimmed from the alignment. The phylogenetic tree was constructed using the maximum likelihood method. **Fig. S3.** Phylogenetic tree based on 18S rRNA gene sequences. Three 18S rRNA genes in the B. caballi genome were compared with representative 18S rRNA genes obtained from other B. caballi specimens and different Babesia species. The sequences are shown with their corresponding GenBank IDs. Clade names are classified according to the nomenclature described by Nehra et al [26]. Numbers on branches represent bootstrap values in the analysis. 

## Data Availability

The genome sequence and annotation are available at DNA Data Bank of Japan (DDBJ; http://www.ddbj.nig.ac.jp/) under accession numbers BPLF01000001-BPLF01000009. Corresponding BioProject and Biosample ID are PRJDB9184 and SAMD00325088, respectively. Nanopore, Illumina, and RNAseq reads are available from SRA under the following accession numbers, DRR394095, DRR296275, and DRR394096, respectively.
