# Supplementary material for: Chromosome-level genome assembly of Babesia caballi reveals diversity of multigene families among Babesia species
Source: BMC Genomics. 2023 Aug 24;24:483. doi: 10.1186/s12864-023-09540-w (PMC10463595; doi:10.1186/s12864-023-09540-w)
Supplement: Supplementary file 2 — Additional file 2: Fig. S1. Structural comparison among apicoplast genomes of Babesia species. The alignments among apicoplast genomes of seven Babesia species are shown using dotplots. Fig. S2. Phylogenetic analysis using apicoplast genomes of seven Babesia species. The genomes were aligned, and gaps were trimmed from the alignment. The phylogenetic tree was constructed using the maximum likelihood method. Fig. S3. Phylogenetic tree based on 18S rRNA gene sequences. Three 18S rRNA genes in the B. caballi genome were compared with representative 18S rRNA genes obtained from other B. caballi specimens and different Babesia species. The sequences are shown with their corresponding GenBank IDs. Clade names are classified according to the nomenclature described by Nehra et al [26]. Numbers on branches represent bootstrap values in the analysis. [file 12864_2023_9540_MOESM2_ESM.pptx]

## Slide 1
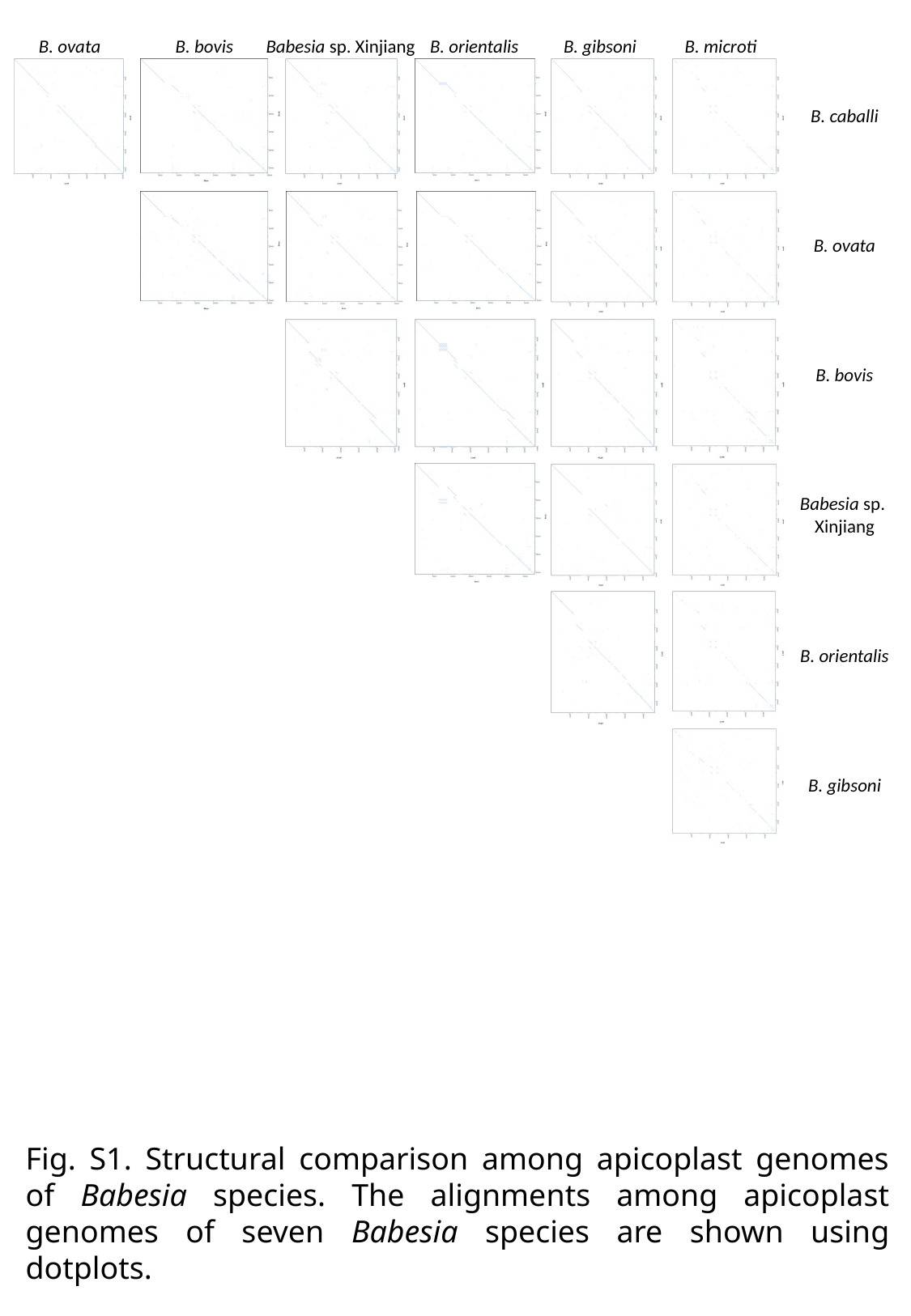

B. ovata
B. bovis
Babesia sp. Xinjiang
B. orientalis
B. gibsoni
B. microti
B. caballi
B. ovata
B. bovis
Babesia sp.
Xinjiang
B. orientalis
B. gibsoni
Fig. S1. Structural comparison among apicoplast genomes of Babesia species. The alignments among apicoplast genomes of seven Babesia species are shown using dotplots.

## Slide 2
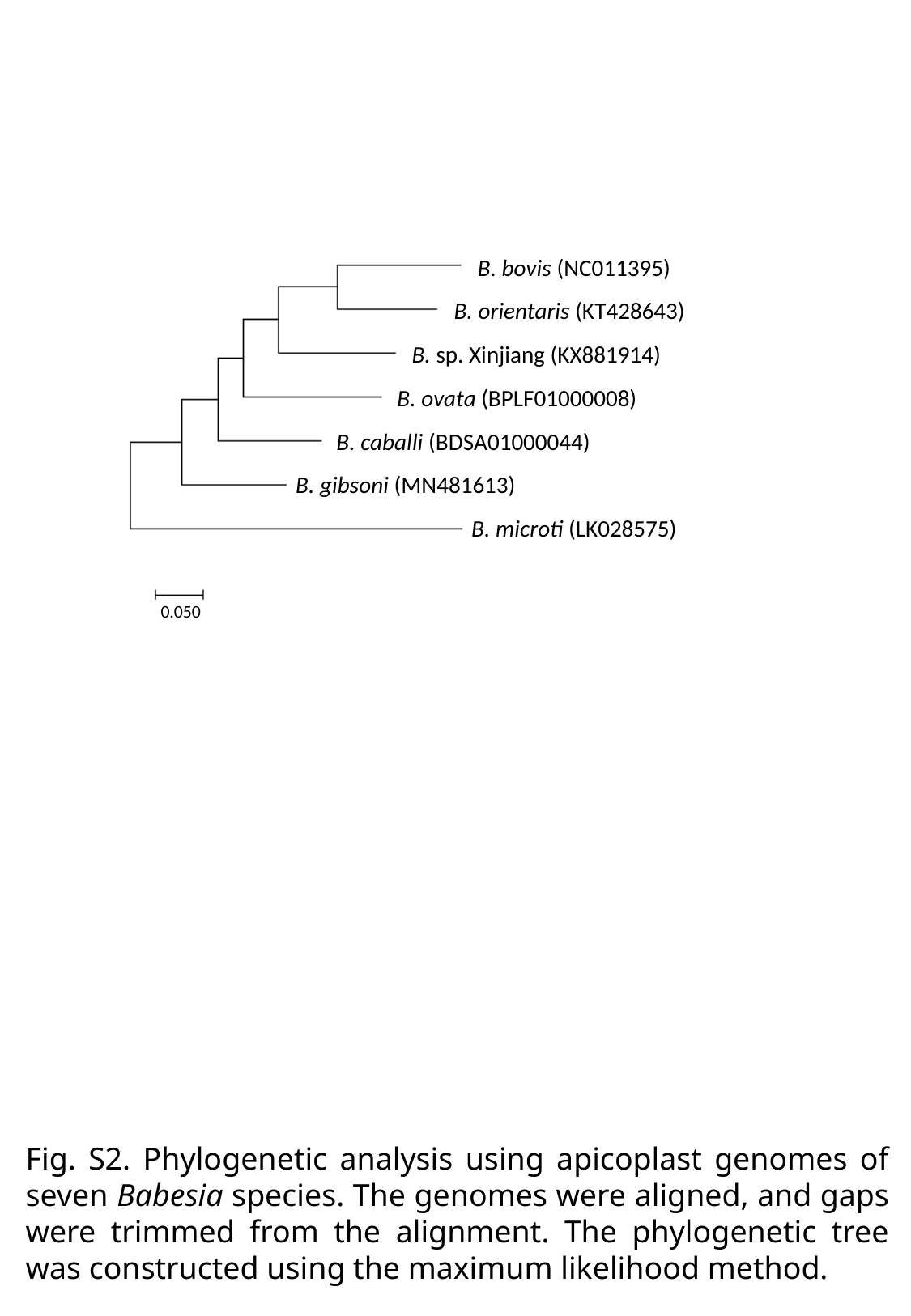

B. bovis (NC011395)
B. orientaris (KT428643)
B. sp. Xinjiang (KX881914)
B. ovata (BPLF01000008)
B. caballi (BDSA01000044)
B. gibsoni (MN481613)
B. microti (LK028575)
0.050
Fig. S2. Phylogenetic analysis using apicoplast genomes of seven Babesia species. The genomes were aligned, and gaps were trimmed from the alignment. The phylogenetic tree was constructed using the maximum likelihood method.

## Slide 3
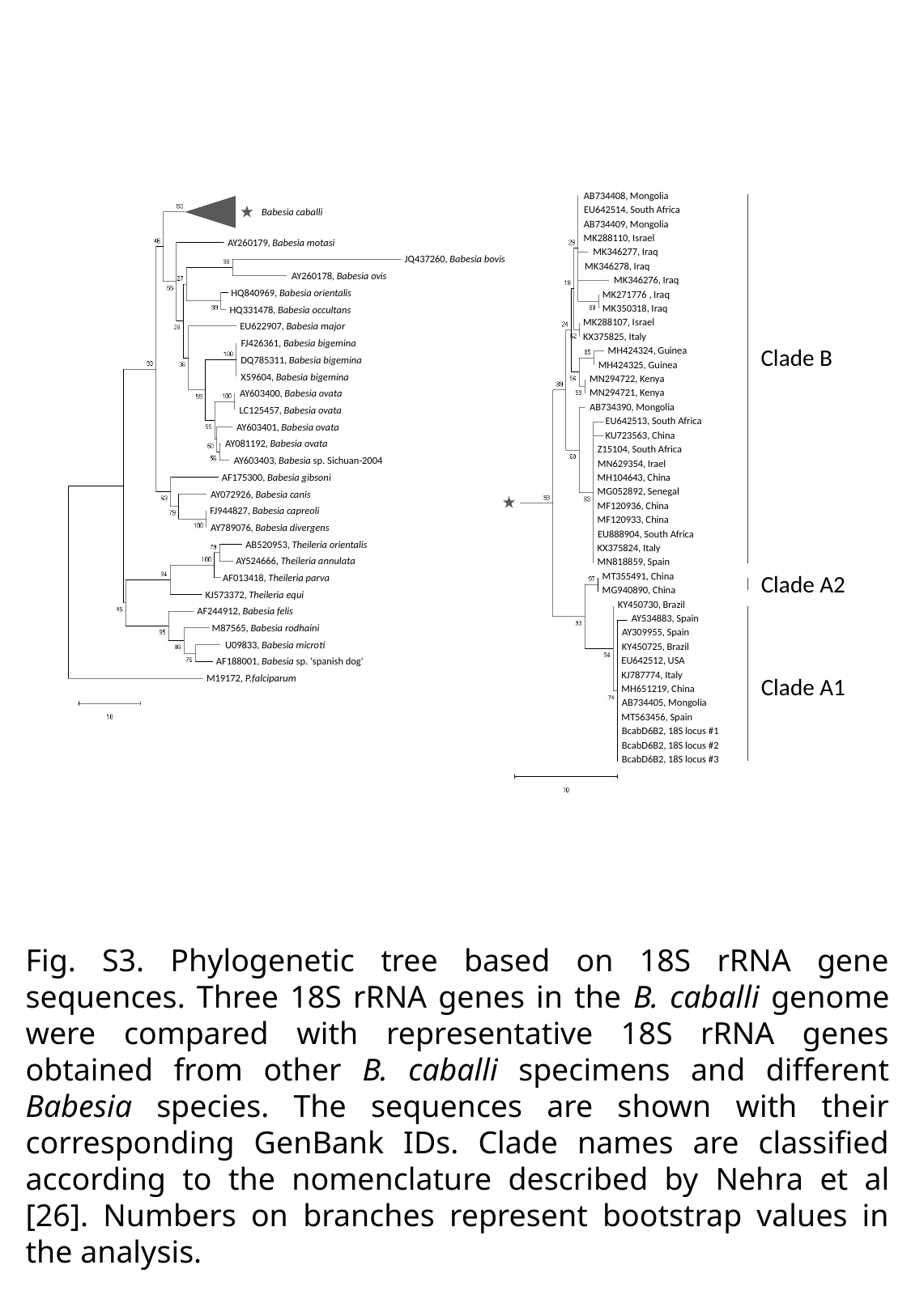

AB734408, Mongolia
EU642514, South Africa
AB734409, Mongolia
MK288110, Israel
MK346277, Iraq
MK346278, Iraq
MK346276, Iraq
MK271776 , Iraq
MK350318, Iraq
MK288107, Israel
KX375825, Italy
Clade B
MH424324, Guinea
MH424325, Guinea
MN294722, Kenya
MN294721, Kenya
AB734390, Mongolia
EU642513, South Africa
KU723563, China
Z15104, South Africa
MN629354, Irael
MH104643, China
MG052892, Senegal
MF120936, China
MF120933, China
EU888904, South Africa
KX375824, Italy
MN818859, Spain
MT355491, China
Clade A2
MG940890, China
KY450730, Brazil
AY534883, Spain
AY309955, Spain
KY450725, Brazil
EU642512, USA
KJ787774, Italy
Clade A1
MH651219, China
AB734405, Mongolia
MT563456, Spain
BcabD6B2, 18S locus #1
BcabD6B2, 18S locus #2
BcabD6B2, 18S locus #3
Babesia caballi
AY260179, Babesia motasi
JQ437260, Babesia bovis
AY260178, Babesia ovis
HQ840969, Babesia orientalis
HQ331478, Babesia occultans
EU622907, Babesia major
FJ426361, Babesia bigemina
DQ785311, Babesia bigemina
X59604, Babesia bigemina
AY603400, Babesia ovata
LC125457, Babesia ovata
AY603401, Babesia ovata
AY081192, Babesia ovata
AY603403, Babesia sp. Sichuan-2004
AF175300, Babesia gibsoni
AY072926, Babesia canis
FJ944827, Babesia capreoli
AY789076, Babesia divergens
AB520953, Theileria orientalis
AY524666, Theileria annulata
AF013418, Theileria parva
KJ573372, Theileria equi
AF244912, Babesia felis
M87565, Babesia rodhaini
U09833, Babesia microti
AF188001, Babesia sp. 'spanish dog'
M19172, P.falciparum
Fig. S3. Phylogenetic tree based on 18S rRNA gene sequences. Three 18S rRNA genes in the B. caballi genome were compared with representative 18S rRNA genes obtained from other B. caballi specimens and different Babesia species. The sequences are shown with their corresponding GenBank IDs. Clade names are classified according to the nomenclature described by Nehra et al [26]. Numbers on branches represent bootstrap values in the analysis.
